# Unraveling robust brain-behavior links of depressive complaints through granular network models for understanding heterogeneity

**DOI:** 10.1016/j.jad.2024.05.060

**Published:** 2024-05-14

**Authors:** René Freichel, Agatha Lenartowicz, Linda Douw, Johann D. Kruschwitz, Tobias Banaschewski, Gareth J. Barker, Arun L.W. Bokde, Sylvane Desrivières, Herta Flor, Antoine Grigis, Hugh Garavan, Andreas Heinz, Rüdiger Brühl, Jean-Luc Martinot, Marie-Laure Paillère Martinot, Eric Artiges, Frauke Nees, Dimitri Papadopoulos Orfanos, Tomáš Paus, Luise Poustka, Nathalie Holz, Christian Baeuchl, Michael N. Smolka, Nilakshi Vaidya, Robert Whelan, Vincent Frouin, Gunter Schumann, IMAGEN Consortium, Henrik Walter, Tessa F. Blanken

**Affiliations:** a Department of Psychology, University of Amsterdam, Amsterdam, the Netherlands; b Semel Institute for Neuroscience and Human Behavior, University of California, Los Angeles, United States; c Department of Anatomy and Neurosciences, Amsterdam University Medical Centers, Vrije Universiteit Amsterdam, Amsterdam, the Netherlands; d Department of Psychiatry and Psychotherapy CCM, Charité – Universitätsmedizin Berlin, Corporate Member of Freie Universität Berlin, Humboldt-Universität zu Berlin, Berlin, Germany; e Department of Child and Adolescent Psychiatry and Psychotherapy, Central Institute of Mental Health, Medical Faculty Mannheim, Heidelberg University, Mannheim, Germany; f Department of Neuroimaging, Institute of Psychiatry, Psychology & Neuroscience, King’s College London, United Kingdom; g Discipline of Psychiatry, School of Medicine and Trinity College Institute of Neuroscience, Trinity College Dublin, Dublin, Ireland; h Social, Genetic and Developmental Psychiatry Centre, Institute of Psychiatry, Psychology and Neuroscience, SGDP Centre, King’s College London, UK; i Institute of Cognitive and Clinical Neuroscience, Central Institute of Mental Health, Medical Faculty Mannheim, Heidelberg University, Mannheim, Germany; j Department of Psychology, School of Social Sciences, University of Mannheim, Mannheim, Germany; k NeuroSpin, CEA, Université Paris-Saclay, Gif-sur-Yvette, France; l Departments of Psychiatry and Psychology, University of Vermont, Burlington, VT, USA; m Physikalisch-Technische Bundesanstalt (PTB), Braunschweig and Berlin, Germany; n Institut National de la Santé et de la Recherche Médicale, INSERM U A10 ‘Trajectoires développementales en psychiatrie’, Université Paris-Saclay, Ecole Normale supérieure Paris-Saclay, CNRS, Centre Borelli, Gif-sur-Yvette, France; o AP-HP, Sorbonne Université, Department of Child and Adolescent Psychiatry, Pitié-Salpêtrière Hospital, Paris, France; p Psychiatry Department, EPS Barthélémy Durand, Etampes, France; q Institute of Medical Psychology and Medical Sociology, Kiel University, Kiel, Germany; r Departments of Psychiatry and Psychology, University of Toronto, Toronto, Ontario, Canada; s Faculty of Psychology, Technische Universität Dresden, Dresden, Germany; t Department of Psychiatry and Neuroimaging Center, Technische Universität Dresden, Dresden, Germany; u Centre for Population Neuroscience and Stratified Medicine (PONS), Department of Psychiatry and Psychotherapy, Charité Universitätsmedizin Berlin, Germany; v School of Psychology and Global Brain Health Institute, Trinity College Dublin, Dublin, Ireland; w Centre for Population Neuroscience and Stratified Medicine (PONS), Department of Psychiatry and Psychotherapy, Charité Universitätsmedizin Berlin, Germany; x Centre for Population Neuroscience and Precision Medicine (PONS), Institute for Science and Technology of Brain-inspired Intelligence (ISTBI), Fudan University, Shanghai, China; y Department of Psychiatry, Faculty of Medicine and Centre Hospitalier Universitaire Sainte-Justine, University of Montreal, Montreal, Quebec, Canada; z Department of Child and Adolescent Psychiatry, Center for Psychosocial Medicine, University Hospital Heidelberg, Heidelberg, Germany

**Keywords:** Depression symptoms, Neural markers, Network analysis, Heterogeneity

## Abstract

**Background:**

Depressive symptoms are highly prevalent, present in heterogeneous symptom patterns, and share diverse neurobiological underpinnings. Understanding the links between psychopathological symptoms and biological factors is critical in elucidating its etiology and persistence. We aimed to evaluate the utility of using symptom-brain network models to parse the heterogeneity of depressive complaints in a large adolescent sample.

**Methods:**

We used data from the third wave of the IMAGEN study, a multi-center panel cohort study involving 1317 adolescents (52.49 % female, mean ± SD age = 18.5 ± 0.7). Two network models were estimated: one including an overall depressive symptom severity sum score based on the Adolescent Depression Rating Scale (ADRS), and one incorporating individual ADRS item scores. Both networks included measures of cortical thickness in several regions (insula, cingulate, mOFC, fusiform gyrus) and hippocampal volume derived from neuroimaging.

**Results:**

The network based on individual item scores revealed associations between cortical thickness measures and specific depressive complaints, obscured when using an aggregate depression severity score. Notably, the insula’s cortical thickness showed negative associations with cognitive dysfunction (partial cor. = − 0.15); the cingulate’s cortical thickness showed negative associations with feelings of worthlessness (partial cor. = − 0.10), and mOFC was negatively associated with anhedonia (partial cor. = − 0.05).

**Limitations:**

This cross-sectional study relied on the self-reported assessment of depression complaints and used a non-clinical sample with predominantly healthy participants (19 % with depression or sub-threshold depression).

**Conclusions:**

This study showcases the utility of network models in parsing heterogeneity in depressive complaints, linking individual complaints to specific neural substrates. We outline the next steps to integrate neurobiological and cognitive markers to unravel MDD’s phenotypic heterogeneity.

## 1. Introduction

Depressive symptoms continue to be highly prevalent across the globe, with increasing rates among adolescents and young people (Goodwin et al., 2022). Depression is a highly heterogeneous disorder (Goldberg, 2011) diagnosed based on the presence of five out of nine DSM-5 symptoms. These symptoms are, however, diverse, ranging from weight loss or gain to depressed mood, and contribute to disorder heterogeneity that poses challenges for treatment. Symptom network models have been used to capture this heterogeneous symptom expression as they conceptualize mental disorders as systems of interacting symptoms. The heterogeneity observed at the level of depression symptoms is mirrored in the disorder’s heterogeneous neurobiological underpinnings (Buch and Liston, 2021): depression has been associated with a wide range of alterations in brain structure and function (Gray et al., 2020; Marx et al., 2023), changes in neurotransmitter systems (Kennis et al., 2020), and genetic variations (Kendall et al., 2021). At the level of neuroanatomical alterations, meta-analytical evidence in adult samples points to lower hippocampal volume (Schmaal et al., 2016) and lower cortical thickness in several regions, including the insula, cingulate, orbitofrontal cortex, and fusiform gyrus (Schmaal et al., 2020). Modeling this interplay between symptom expression and biology is crucial for understanding depression’s etiology and, ultimately, treatment (Remes et al., 2021).

However, when both domains (i.e., psychological/biological) are combined, then typically, at least one domain is simplified in the process (Blanken et al., 2021), often to a single aggregate dimension. Most studies examining associations between structural and functional neural alterations and depressive symptoms, either use depression sum scores or subscales (aggregating the psychological level) or they use aggregate measures derived from neuroimaging, such as overall cortical thickness, or structural or functional connectivity (aggregating the biological level). This abstraction potentially obscures more fine-grained associations, that could potentially account for the symptom heterogeneity.

While many studies have revealed close relationships between depression and brain structure and function (e.g., Schmaal et al., 2020), fewer studies have examined this link for specific depressive complaints. For instance, social anhedonia symptoms have been associated with reduced (gray) matter volume in the bilateral caudate nucleus (Enneking et al., 2019). Similarly, there is evidence for associations between disturbed white matter microstructure and cognitive dysfunction in depression (Meinert et al., 2022). One recent pilot study that included both brain and individual symptom measures into one network model did reveal cross-modal (i.e., brain-symptom) relations even in a small sample of depressed and never-depressed adults (Hilland et al., 2020). This finding suggests that fine-grained associations could indeed be obscured when using aggregate measures, but this was not evaluated directly.

We believe that network analysis (Borsboom et al., 2021) offers distinct advantages for studying granular cross-modal associations between individual symptoms and brain markers. First, network analysis can identify unique, conditional (i.e., partial) associations while controlling for the influence of all other symptom nodes or brain markers in the model. Given the many and strong relations between the depressive symptoms themselves, this provides the opportunity to distinguish direct from indirect effects. Second, from a conceptual perspective, network analysis maps onto the complex organization of mental disorders that consists of interconnected symptoms, cognitive, and neurobiological features (Blanken et al., 2021).

In the present study, we replicate the approach by Hilland et al. (2020) in a substantially larger sample to identify relations between depressive complaints and five a-priori selected (based on Hilland et al., 2020) brain markers (cortical thickness measures for insula, cingulate, mOFC, fusiform gyrus, and hippocampal volume). In addition, we will extend the previous study by directly evaluating whether parsing heterogeneity into individual item scores relative to an overall severity measure reveals cross-modal relations that otherwise would remain hidden.

## 2. Methods

### 2.1. Participants, procedure, and outcomes

We have used data from the third wave of IMAGEN study (Schumann et al., 2010), a multi-center panel cohort study of adolescents. Our final sample included 1317 adolescents (52.49 % female, M ± SD = 18.5 ± 0.7 years old, range: 18–23 years old) that completed the Adolescent Depression Rating Scale (ADRS) and 3D T1-weighted gradient-echo Magnetic Resonance Imaging (MRI) scans. The ADRS is a validated 10-item self-report scale to assess the presence (present/not present) of adolescent depression symptoms (Revah-Levy et al., 2007). The scale consists of 10 items that assess the presence of different depressive complaints on a binary scale (1 = *True*/*Present*, 0 = *False*/*Not present*). A total ADRS depression severity (sum) score above 6 is commonly used as a cut-off for a clinically relevant diagnosis of MDD as it ensures maximum sensitivity and specificity (Revah-Levy et al., 2007, 2011; Vulser et al., 2015). ADRS scores of 3 to 5 indicate “sub-threshold depression” (Revah-Levy et al., 2011).

**Fig. 1.**
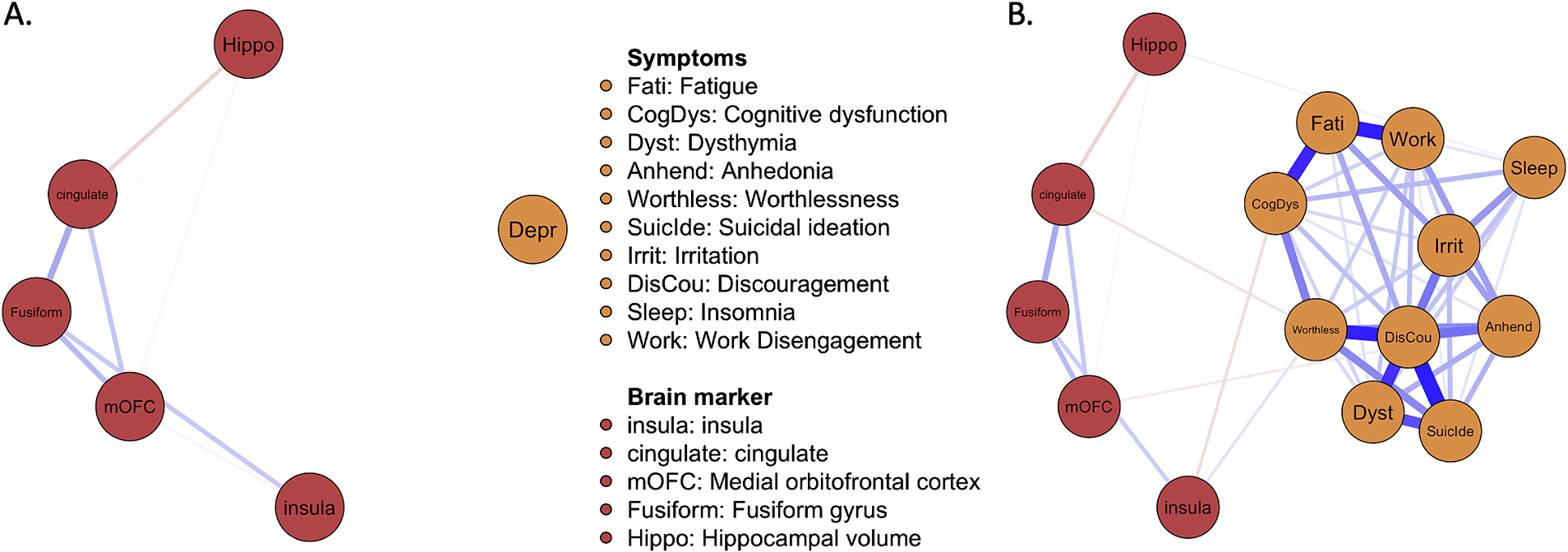
Depressive complaints — brain network model. Note. The thickness of the lines indicates the strength of association. The connections (edges) in the network represent pairwise, partial associations between different complaints and brain markers. Positive conditional associations are colored in blue, negative conditional associations are colored in red. Panel A includes the ADRS severity score (*Depr*). Panel B includes all ADRS depression complaints. The nodes for the four brain regions (i.e., insula, cingulate, mOFC, Fusiform) refer to cortical thickness. *Hippo* = hippocampal volume; *mOFC* = medial orbitofrontal cortex. All edge weights can be found in supplementary Tables S2–S3. The cut argument has been set to 0. Both networks were visualised using the same maximum edge weight for scaling. (For interpretation of the references to color in this figure legend, the reader is referred to the web version of this article.)

The present sample showed substantial variability in the presence of all complaints (see Table S1 in supplementary materials (SM) section 1), with 12 % (*n* = 155) of individuals being in the ‘sub-threshold’ depression group, and an additional 7 % of individuals (*n* = 89) meeting the criteria (score ≥ 6) for MDD. The magnetic resonance imaging (MRI) data was acquired using standard protocols to ensure homogeneity across scanners, including a 3D T1-weighted gradient echo volume (see SM1 and Schumann et al., 2010 for more details). Cortical thickness of insula, cingulate, mOFC, fusiform and hippocampal volume were estimated using the FreeSurfer software. We selected the same five brain regions (see Fig. 1) as Hilland et al. (2020) and followed their exact procedures: we averaged left/right hemispheres and used z-residuals for hippocampal volume (regressing out sex, intracranial volume). Age was not included as a covariate in the model considering that our sample was based on one assessment wave from a cohort study comprising adolescents of a comparable age group, with an average age of 18.5 years and a standard deviation of 0.7 years.

### 2.2. Statistical analysis

To investigate whether the abstraction of symptoms as sum scores obscures more fine-grained relations between brain regions and depression complaints, we estimated two network models. Both networks contained the same brain measures (i.e., cortical thickness measures, hippocampal volume); however, one included the ADRDS sum score, indicating overall depression severity, and one included all individual ADRS items, representing different depressive complaints. The network estimation includes a nodewise regression approach in which every node is predicted by all other nodes. To minimize false positive edges, we have used LASSO (Least Absolute Shrinkage and Selection Operator) regularization with cross-validation (determines optimal level of penalization) to shrink estimates towards zero and avoid overfitting (see SM2 for more details). For the network including depression severity, we estimated a Gaussian Graphical Model, including all variables as continuous, whereas for the network including the single depression items, we estimated a Mixed Graphical Model, including the items as binary variables (present/not present) and the brain measures as continuous, taking the different variable types into account (MGM, Haslbeck and Waldorp, 2020). The resulting connections in both networks (‘edges’) represent pairwise conditional associations (similar to partial correlations) that control for all other nodes in the network. While traditional statistical significance is not defined in these models, edges are included based on model fit. Included edges thus improve the fit of the model to the data. We assessed the edge weights’ accuracy using bootstrapping (*n* = 1000, see SM2).

## 3. Results

The network including depression severity is shown in Fig. 1A. We found no cross-modal associations between any of the neural markers and overall depression severity. In contrast, we found many positive associations within the respective domains (i.e., among depressive complaints and cortical thickness measures) in the network estimated on the separate depression complaints (Fig. 1B). Interestingly, we found cross-modal associations between cortical thickness measures and specific complaints: cingulate was negatively associated with *worthlessness* (retrieved in 59 %), insula was negatively associated with *cognitive dysfunction* (85 % retrieved), and mOFC was negatively associated with *anhedonia* (53 % retrieved). We found positive associations between insula and *worthlessness* (61 % retrieved) and between hippocampal volume and *sleep* problems (60 % retrieved). The networks were sufficiently stable, and all cross-modal links were retrieved in at least half of the bootstrapped samples (range 53–85 %). An additional subgroup analysis of individuals with either sub-threshold depression or meeting all criteria for a depression diagnosis showed differences but replicated two cross-modal associations (*worthlessness* — cingulate; insula — worthlessness).

## 4. Discussion

The present study is one of the first to pinpoint granular associations between neural substrates of overall depressive symptomatology and specific depression complaints using an integrated network approach. Crucially, we showed that these robust associations remain hidden when only including overall depression severity, concealing the heterogeneous complaints. The negative associations shown (between regional cortical thickness and complaints) align with prior evidence for cortical thinning as a depression biomarker (Suh et al., 2019), prompting us to speculate about the mechanisms at play. The link between cortical thinning of the insula and cognitive dysfunction could reflect the insula’s pivotal role in high-level cognitive control and emotional processing (Menon and Uddin, 2010). This interpretation about altered affective processing in depression may be particularly relevant, in light of our findings regarding the negative association between cingulate’s cortical thickness and feelings of worthlessness, given the prominent role of the cingulate cortex in emotional processing (Etkin et al., 2011). At the same time, we want to stress that such interpretations are highly speculative, as our estimated links are undirected and estimated cross-sectionally. Interestingly, our results also uncovered novel links, such as positive associations between *insula* and *worthlessness*.

We believe that our findings have dual implications; with respect to guiding future brain-behavior research and bear relevance for clinical practice. First, our comparative analysis of networks estimated on an aggregate measure of depression severity (Fig. 1A) and specific depression complaints (Fig. 1B) showed stark differences. The heterogeneity underlying the association between neural substrates and depressive complaints was obscured when using an aggregate score. This suggests that networks estimated at the level of individual symptoms and neural makers have the potential to dissect these hidden associations and may allow us to better grasp the heterogeneity of depression.

Second, while primarily exploratory, symptom-brain networks, as showcased in this report, may inspire research that could eventually be used for potential clinical applications. The current diagnostic heterogeneity of depression complicates an effective treatment (Buch and Liston, 2021). Thus, identifying specific symptom-brain biomarker connections, such as the link between the thinning of insula and cognitive dysfunction, may pave the way for delineating distinct psychobiological subtypes of depression. This may potentially lead to more accurate diagnostic and tailored treatment approaches, moving us closer to a personalized medicine model in psychiatry. However, this is a more conceptual point as we refrain from drawing a direct line from a cross-sectional association study in a particular sample to real-world clinical applications.

### 4.1. Limitations

A limitation of our study is the self-reported assessment of depressive complaints that may naturally be biased. In addition, our sample was relatively healthy (only 19 % of participants with depression or sub-threshold depression), and thus, the cross-modal links should be understood as associations describing how variability in depressive complaints is linked to variability in the selected brain markers. Future studies should replicate our findings using clinical samples with a higher number of individuals with MDD. Lastly, the cross-sectional nature of our study precludes any conclusions about the directionality and causal nature of the associations between neural markers and depressive complaints. The present study serves as a ‘proof-of-principle’ that may inspire future work to validate the mapping of symptoms and neural markers in clinical samples.

## 5. Conclusions

Altogether, this brief report showcases the utility of brain-symptom networks in the case of depressive complaints. Moving forward, future research should adopt such approaches and integrate neurobiological and cognitive markers to parse the phenotypic heterogeneity of depressive symptomatology both at a cross-sectional and developmental level.

## Supplementary Material

SupplementaryMaterial
