# Supplementary material for: Unraveling robust brain-behavior links of depressive complaints through granular network models for understanding heterogeneity
Source: J Affect Disord. Author manuscript; Available in PMC 2024 Dec 31. (PMC11687178; doi:10.1016/j.jad.2024.05.060)
Supplement: SupplementaryMaterial [file NIHMS2041067-supplement-SupplementaryMaterial.docx]

**Unraveling robust brain-behavior links of depressive complaints through granular network models for understanding heterogeneity**

**Supplementary Materials**

Supplementary Material Section 1: Study and Network Descriptives 2

Supplementary Material Section 2: Network analysis and stability 6

Supplementary Material Section 3: Subgroup analysis 10

# Supplementary Material Section 1: Study and Network Descriptives

**Neuroimaging**

The neuroimaging procedure is described in detail elsewhere (Schumann et al., 2010; Vulser et al., 2015). Total intracranial volume and sex were used as covariates when estimating hippocampal volume following the procedure by Hilland et al. (2020). Total intracranial volume was based on the summation of gray matter, cerebrospinal fluid (CSF), and white matter volumes, and this measure was used in prior work on this dataset (Quinlan et al., 2020; Vulser et al., 2015). MR data quality-control showed that Total Intracranial Volumes (TIVs) change up to 25% in some participants from one evaluation wave to another. This suggests that there might be measurement or segmentation errors that could affect the statistical results.

**ADRS depression measurement**

The internal consistency of the ADRS scale in our data was good (Cronbach’s alpha based on Kuder and Richardson formula for dichotomous items: 0.83). There was substantial variability in the symptom presence (see Table S1) and the aggregate depression severity score (*M =* 1.29, *SD* = 2.09).

**Table S1**

*Proportion of ADRS Symptom Presence*

| **ADRS Item** | **n nymptom present** | **Proportion symptom present** |
| --- | --- | --- |
| Fati | 256 | 19.44% |
| CogDys | 161 | 12.22% |
| Dyst | 174 | 13.21% |
| Anhend | 75 | 5.69% |
| Worthless | 111 | 8.43% |
| SuicIde | 90 | 6.83% |
| Irrit | 174 | 13.21% |
| DisCou | 205 | 15.57% |
| Sleep | 311 | 23.61% |
| Work | 147 | 11.16% |

*Note. Fati = Fatigue, CogDys = Cognitive dysfunction, Dyst = Dysthymia, Anhend = Anhedonia, Worthless = Worthlessness, SuicIde = Suicidal ideation, Irrit = Irritation, DisCou = Discouragement, Sleep = Insomnia, Work = Work Disengagement.*

**Table S2**

*Edge Weights for Brain-Depression Sum Score Network Model (see Figure 1A)*

|  | **Depr** | **insula** | **cingulate** | **mOFC** | **Fusiform** | **Hippo** |
| --- | --- | --- | --- | --- | --- | --- |
| **Depr** |  |  |  |  |  |  |
| **insula** | 0 |  |  |  |  |  |
| **cingulate** | 0 | 0 |  |  |  |  |
| **mOFC** | 0 | 0.03 | 0.22 |  |  |  |
| **Fusiform** | 0 | 0.21 | 0.32 | 0.26 |  |  |
| **Hippo** | 0 | 0 | -0.17 | -0.03 | -0.02 |  |

*Note.* Depr = Depression ADRS sum score, Hippo = Hippocampal volume, mOFC = Medial Orbitofrontal Cortex cortical thickness, Fusiform = Fusiform Gyrus cortical thickness, Insula = Insula cortical thickness, Cingulate = Cingulate cortical thickness.

**Table S3**

*Edge Weights for Brain-Symptom Network Model (see Figure 1B)*

|  | **Fati** | **CogDys** | **Dyst** | **Anhend** | **Worthless** | **SuicIde** | **Irrit** | **DisCou** | **Sleep** | **Work** | **insula** | **cingulate** | **mOFC** | **Fusiform** |
| --- | --- | --- | --- | --- | --- | --- | --- | --- | --- | --- | --- | --- | --- | --- |
| **Fati** |  |  |  |  |  |  |  |  |  |  |  |  |  |  |
| **CogDys** | 0.8 |  |  |  |  |  |  |  |  |  |  |  |  |  |
| **Dyst** | 0.11 | 0.17 |  |  |  |  |  |  |  |  |  |  |  |  |
| **Anhend** | 0 | 0.11 | 0.32 |  |  |  |  |  |  |  |  |  |  |  |
| **Worthless** | 0 | 0.5 | 0.14 | 0.44 |  |  |  |  |  |  |  |  |  |  |
| **SuicIde** | 0 | 0 | 0.67 | 0.28 | 0.44 |  |  |  |  |  |  |  |  |  |
| **Irrit** | 0.37 | 0.16 | 0.38 | 0.37 | 0.21 | 0.26 |  |  |  |  |  |  |  |  |
| **DisCou** | 0.32 | 0.24 | 0.76 | 0.54 | 0.85 | 0.95 | 0.52 |  |  |  |  |  |  |  |
| **Sleep** | 0 | 0.29 | 0.2 | 0 | 0 | 0.09 | 0.41 | 0.23 |  |  |  |  |  |  |
| **Work** | 0.89 | 0.19 | 0.22 | 0.37 | 0.19 | 0.07 | 0.19 | 0.25 | 0.1 |  |  |  |  |  |
| **insula** | 0 | -0.15 | 0 | 0 | 0.12 | 0 | 0 | 0 | 0 | 0 |  |  |  |  |
| **cingulate** | 0 | 0 | 0 | 0 | -0.1 | 0 | 0 | 0 | 0 | 0 | 0 |  |  |  |
| **mOFC** | 0 | 0 | 0 | -0.05 | 0 | 0 | 0 | 0 | 0 | 0 | 0 | 0.21 |  |  |
| **Fusiform** | 0 | 0 | 0 | 0 | 0 | 0 | 0 | 0 | 0 | 0 | 0.19 | 0.32 | 0.26 |  |
| **Hippo** | 0 | 0 | 0 | 0 | 0 | 0 | 0 | 0 | 0.04 | 0 | 0 | -0.16 | 0 | 0 |

*Note.* Fati = Fatigue, CogDys = Cognitive dysfunction, Dyst = Dysthymia, Anhend = Anhedonia, Worthless = Worthlessness, SuicIde = Suicidal ideation, Irrit = Irritation, DisCou = Discouragement, Sleep = Insomnia, Work = Work Disengagement, Hippo = Hippocampal volume, mOFC = Medial Orbitofrontal Cortex cortical thickness, Fusiform = Fusiform Gyrus cortical thickness, Insula = Insula cortical thickness, Cingulate = Cingulate cortical thickness

# Supplementary Material Section 2: Network analysis and stability

**Network estimation.**

Before estimating the network, we checked for potential multicollinearity among all included variables. The mean zero-order correlation (among all variables included in the network) was 0.16, and the maximum zero-order correlation was 0.53. This did not indicate concerns regarding multicollinearity in the network estimation.

Cross-validation (with 10 folds) was used to select the tuning parameter parameter *lambda* in L1 (LASSO) regularization. In every iteration of the 10-fold cross-validation, the dataset is divided into 10 subsets. Nine of these subsets are used to train the model, and the remaining one is used to test the model. This process is repeated 10 times, with each subset serving as the validation set once. Throughout these iterations, the optimal lambda value for regularization is determined by identifying the lambda that minimizes the mean cross-validated error. This process reduces the risk of overfitting and increases the robustness of the estimation. More information on this estimation procedure can be found in the documentation of the *mgm* package (Haslbeck & Waldorp, 2020).

Cross-validation provides sufficient sensitivity and was considered appropriate as we were interested in identifying cross-modal links that may not be detected when using more stringent procedures for penalization, such as the Extended Bayesian Information Criterion (EBIC). We have used the Fruchterman-reingold algorithm (Fruchterman & Reingold, 1991) to determine the layout of the item-level network visualization. To facilitate visual comparison, we have used the same maximum edge strength for scaling the edge weights in both the item- and sum score networks.

**Network stability.**

To examine the stability of the estimated networks, we have used a non-parametric bootstrapping approach (Epskamp et al., 2018). This re-estimates the model under sampled data (with replacement) 1000 times. A bootstrapped confidence interval around the edge weights (see Figure S1) is calculated and describes the accuracy of the edge weights. See Epskamp et al. (2018) for a tutorial on non-parametric bootstrapping.

**Figure S1**

*Non-Parametric Bootstrap for Brain-Depression Sum Score Network Model (see Figure 1A)*

*
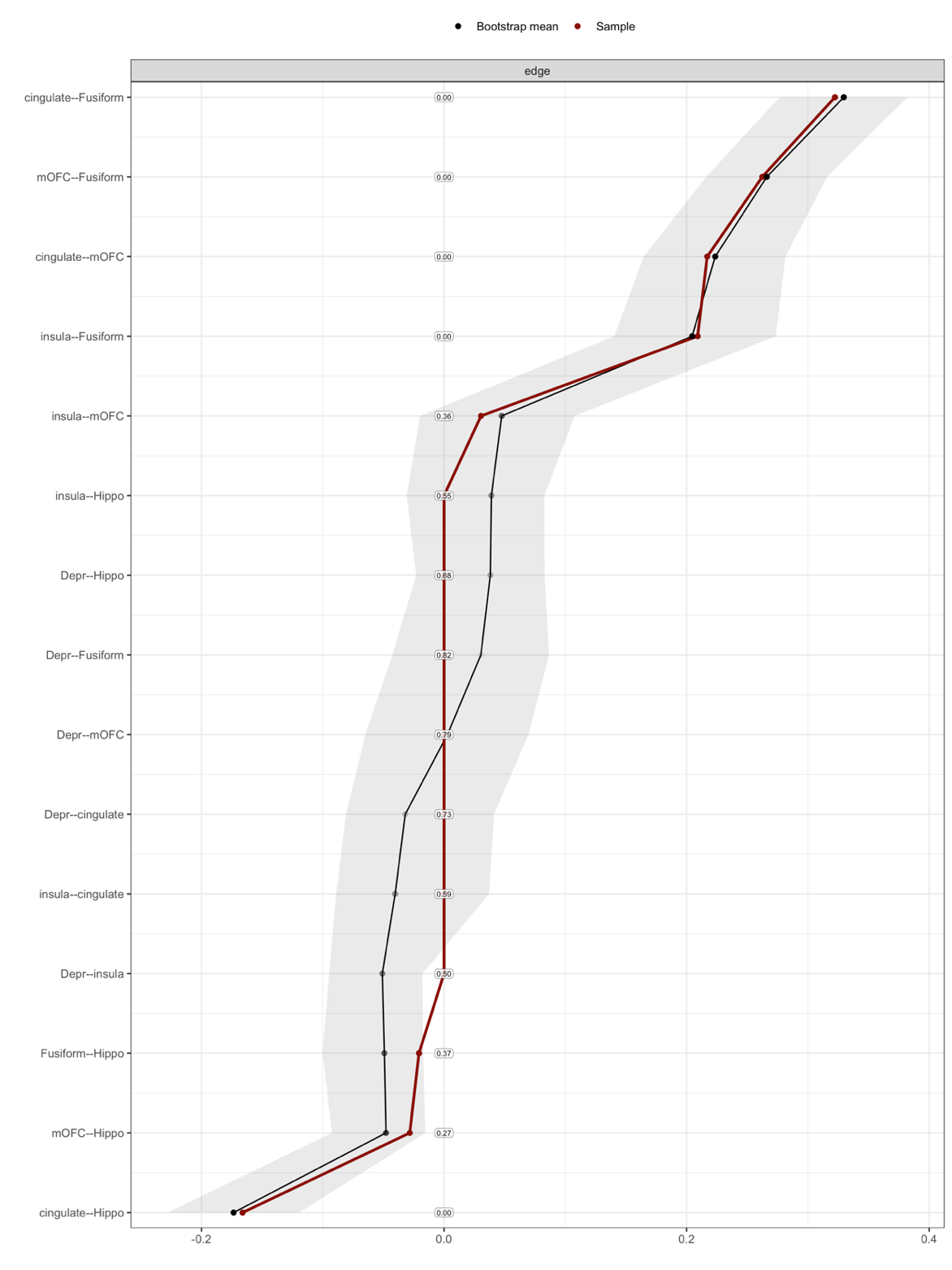
*

*Note.* The grey area describes the 95% bootstrapped confidence interval of the estimated edge weights. The sample values are shown in red. The edges are ordered according to their weight from high (top) to low (bottom). The proportion of estimates being zero is shown in the middle.

**Figure S2**

*Non-parametric Bootstrap for Brain-Depression Symptom Network Model (see Figure 1B)*


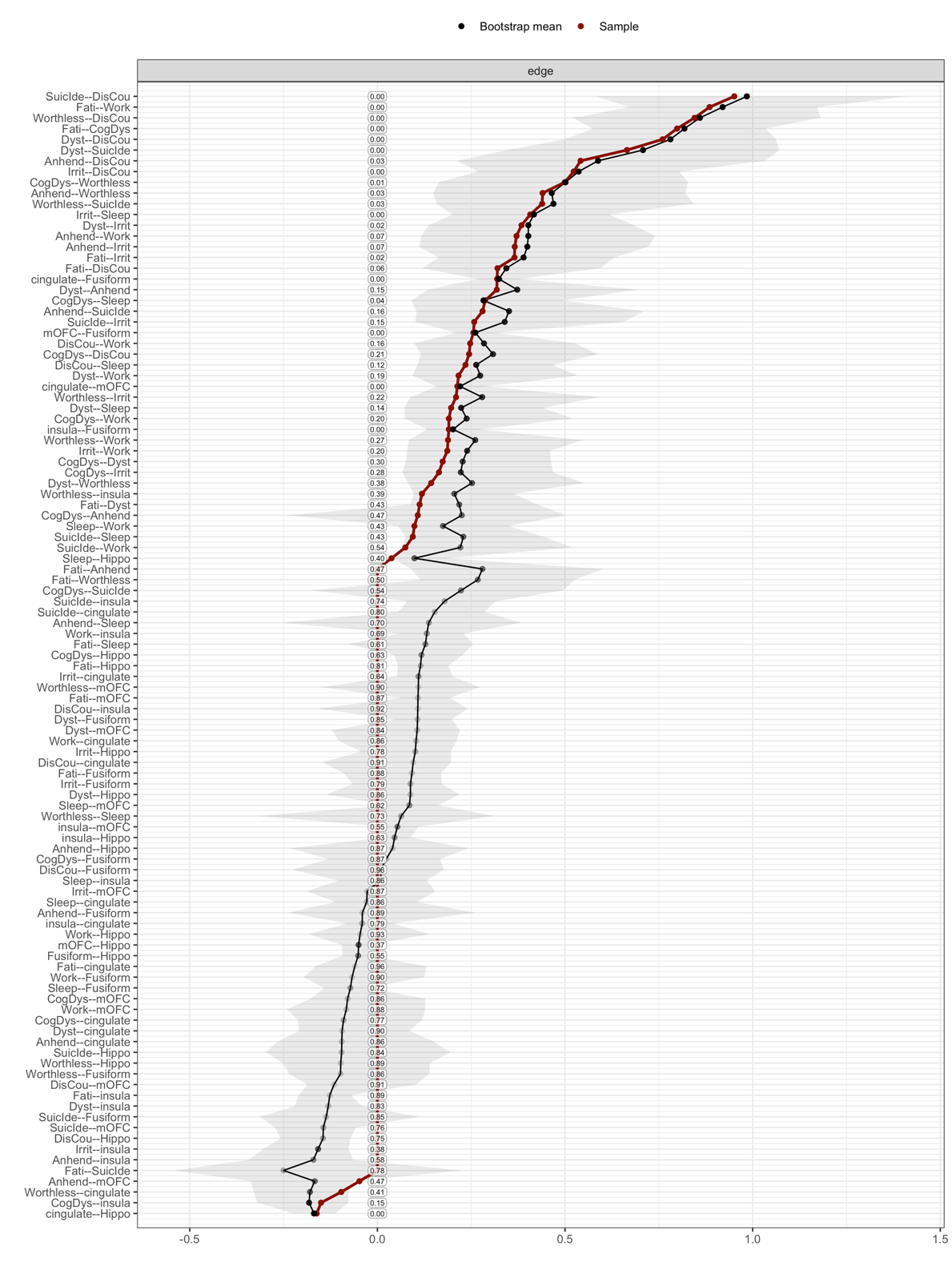


*Note.* The grey area describes the 95% bootstrapped confidence interval of the estimated edge weights. The sample values are shown in red. The edges are ordered according to their weight from high (top) to low (bottom). The proportion of estimates being zero is shown in the middle.

# Supplementary Material Section 3: Subgroup analysis

**Figure S3**

*Depressive Complaints – Brain Marker Network Model In Individuals With (Sub-threshold) Depression*


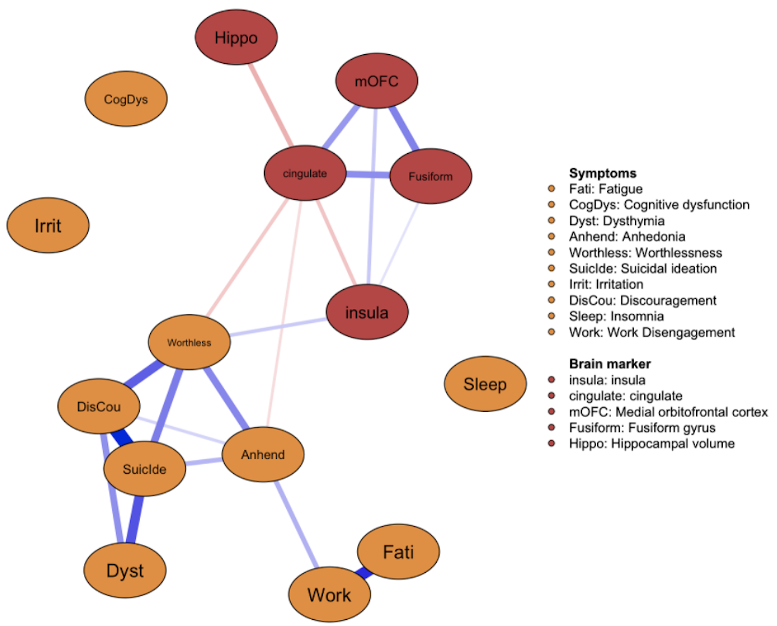


**References**

Epskamp, S., Borsboom, D., & Fried, E. I. (2018). Estimating psychological networks and their accuracy: A tutorial paper. *Behavior Research Methods*, *50*(1), 195–212. https://doi.org/10.3758/s13428-017-0862-1

Fruchterman, T. M. J., & Reingold, E. M. (1991). Graph drawing by force‐directed placement. *Software: Practice and Experience*, *21*(11), 1129–1164. https://doi.org/10.1002/spe.4380211102

Haslbeck, J. M. B., & Waldorp, L. J. (2020). Mgm: Estimating time-varying mixed graphical models in high-dimensional data. *arXiv:1510.06871 [Stat]*. http://arxiv.org/abs/1510.06871

Hilland, E., Landrø, N. I., Kraft, B., Tamnes, C. K., Fried, E. I., Maglanoc, L. A., & Jonassen, R. (2020). Exploring the links between specific depression symptoms and brain structure: A network study. *Psychiatry and Clinical Neurosciences*, *74*(3), 220–221. https://doi.org/10.1111/pcn.12969

Quinlan, E. B., Barker, E. D., Luo, Q., Banaschewski, T., Bokde, A. L. W., Bromberg, U., Büchel, C., Desrivières, S., Flor, H., Frouin, V., Garavan, H., Chaarani, B., Gowland, P., Heinz, A., Brühl, R., Martinot, J.-L., Martinot, M.-L. P., Nees, F., Orfanos, D. P., … Schumann, G. (2020). Peer victimization and its impact on adolescent brain development and psychopathology. *Molecular Psychiatry*, *25*(11), Article 11. https://doi.org/10.1038/s41380-018-0297-9

Revah-Levy, A., Birmaher, B., Gasquet, I., & Falissard, B. (2007). The Adolescent Depression Rating Scale (ADRS): A validation study. *BMC Psychiatry*, *7*, 2. https://doi.org/10.1186/1471-244X-7-2

Schumann, G., Loth, E., Banaschewski, T., Barbot, A., Barker, G., Büchel, C., Conrod, P. J., Dalley, J. W., Flor, H., Gallinat, J., Garavan, H., Heinz, A., Itterman, B., Lathrop, M., Mallik, C., Mann, K., Martinot, J.-L., Paus, T., Poline, J.-B., … Struve, M. (2010). The IMAGEN study: Reinforcement-related behaviour in normal brain function and psychopathology. *Molecular Psychiatry*, *15*(12), Article 12. https://doi.org/10.1038/mp.2010.4

Vulser, H., Lemaitre, H., Artiges, E., Miranda, R., Penttilä, J., Struve, M., Fadai, T., Kappel, V., Grimmer, Y., Goodman, R., Stringaris, A., Poustka, L., Conrod, P., Frouin, V., Banaschewski, T., Barker, G. J., Bokde, A. L. W., Bromberg, U., Büchel, C., … Stephens, D. (2015). Subthreshold Depression and Regional Brain Volumes in Young Community Adolescents. *Journal of the American Academy of Child & Adolescent Psychiatry*, *54*(10), 832–840. https://doi.org/10.1016/j.jaac.2015.07.006
